# Comparison of two anorganic bovine bone in maxillary sinus lift: a split-mouth study with clinical, radiographical, and histomorphometrical analysis

**DOI:** 10.1186/s40729-020-00214-w

**Published:** 2020-05-06

**Authors:** Heitor Fontes da Silva, Douglas Rangel Goulart, Alexander Tadeu Sverzut, Sergio Olate, Márcio de Moraes

**Affiliations:** 1 Division of Oral and Maxillofacial Surgery, Department of Oral Diagnosis, Piracicaba Dental School, Campinas State University Unicamp, Av. Limeira, 901, Areiao, Piracicaba, São Paulo 13414-903 Brazil; 2 Dental School University of Goiás — UFG, Goiânia, Brazil; 3 Division of Oral and Maxillofacial Surgery, Department of Oral Diagnosis, Piracicaba Dental School, Campinas State University—Unicamp, São Paulo, Brazil; 4 Department of Oral, Facial and Maxillofacial Surgery and Center of Excellence in Surgical and Morphological Studies, University of La Frontera, Temuco, Chile

**Keywords:** Maxillary sinus, Dental implants, Sinus floor elevation, Anorganic bovine bone, Bone grafting, Clinical trial

## Abstract

**Background:**

Anorganic bovine bone (Bio-Oss®) has been extensively used for reconstruction of posterior area of maxilla in sinus lift procedure; however, a new graft material (Lumina-Bone Porous®), that has a different manufacturing process, has not been yet compared in clinical and histological terms. The manufacturing process of bovine bone graft is related to size and porosity of the particles, and this can change osteoconductive property of the material and bone formation. The use of Lumina-Porus® could improve bone formation, reduce the remaining particles of the biomaterial using a low-cost material. The aim of this research was to compare the clinical, radiological, and histomorphometrical results from maxillary sinus lift with two different anorganic bovine bone substitutes Bio-Oss® (control) and Lumina-Bone Porous® (test).

**Results:**

A split-mouth study was performed with 13 volunteers. The mean bone ridge height in the deepest portion of maxillary sinuses floor was 3.11 ± 0.83 mm in the Bio-Oss® and 2.38 ± 0.75 mm in the Lumina-Bone Porous®. After sinus lift, the Bio-Oss® group shows bone ridge height of 11.56 ± 2.03 mm and Lumina-Bone® of 10.62 ± 1.93 mm. The increase in alveolar bone height scores was significant between pre-augmentation and 6 months after SL in both groups (*p* < 0.001). No statistical significant difference in newly formed bone in the Bio-Oss® group (20.4 ± 5.4%), and Lumina-Bone Porous® (22.8 ± 8.5%) was histomorphological observed (*p* > 0.05). On the other hand, the residual graft particles showed significant difference between the Bio-Oss® group (19.9 ± 8.6%) and Lumina-Bone Porous® (14.6 ± 5.6%) (*p* < 0.05). The survival rate of dental implants for augmented area with Lumina Bone Porous® was 88.88%, while for Bio-Oss® group was 100%.

**Conclusion:**

Both materials Bio-Oss® and Lumina-Bone Porous® can be used in the maxillary sinus floor augmentation with good predictability in clinical, radiographical, and histological point of view.

## Introduction

Dental implants are the gold standard treatment for replacing missing teeth as a support for dental prostheses to obtain aesthetic and function. In the posterior area of maxilla, the loss of alveolar bone height and bone density related to maxillary sinus is a challenge for surgeons [1]. Boyne and James published the first report about “sinus lift procedure” in 1980, and this technique has been studied several times over the years. The lateral window approach is a commonly used technique for maxillary sinus floor augmentation [2].

Sinus lift procedure is a surgical technique aimed to increasing the height of residual bone in the posterior maxilla by repositioning the floor of maxillary sinus in upward direction, creating appropriated bone height that can accommodate appropriately the placement of functional dental implants [3]. Alternative treatment could be performed with tilted implants (angulated direction that avoid maxillary sinus), zygomatic implants, and more recently short implants (4 to 8 mm long) [1].

Autogenous bone is still considered by many surgeons as the most predictable material for bone augmentations, due to their properties as osteogenic, osteoconductive, and osteoinductive [4]. The disadvantages of autogenous bone are the limited amount of available, donor site morbidity, and more surgical time [5].

During the last decade, a number of new bone substitutes have been introduced to the market. Among natural biomaterials, xenografts from mammal species are promising due to similarities in bone architecture and collagen composition [6]. Tissues from different species have been used, mainly bovine, swine, and equine bone. Thermo-chemical process removes organic component and could create a mineral scaffold with residual collagen. These could be delivered in bone particles or blocks [1]. Deproteinized bovine bone (DBB) includes of 100% anorganic bovine bone, show to be safe and biocompatible material with osteoconductive properties [7].

Histomorphometric results in the augmented sinus are not associated to survival rate of the implants; however, it is a reliable tool to asses and compare the graft materials [8]. There are several xenografts derived from bovine bone, currently in use for clinical practice with support of evidence of bone formation and clinical success [6, 9, 10]. According with the process, it is possible that some material from similar origin could be act in different form and the requirement is to analyze this condition from multiple methodologies. In this sense, clinical, radiographical, and histomorphometrical comparison is necessary.

Lumina-Bone Porous® is a material produced from bovine inorganic bone. Its manufacturing process is sinter-free and presents a chemical sterilization. The chemical process maintains the collagen chain and promotes porosities in 75% of the surface of the particles. This can improve the osteoconductive property, which can lead to clinical results equal to or greater than the reference material. In addition, this material has national technology and manufacturing, which represents cost reduction. The aim of this research is to compare the performance of two different xenografts in a split-mouth model using the sinus lift technique. The null hypothesis is that there is no difference between the materials tested from a clinical point of view (number of implants lost) and histological (area of newly formed bone and area residual graft particles). The alternative hypothesis is that one material performed better than the other in the clinical and/or histological evaluation.

## Methods

### Study design and randomization

A split-mouth study was performed to compare two xenograft materials in the sinus lift techinque using lateral approach. The choice of whether the sinus (left or right) would contain the test biomaterial (Lumina-Bone Porous®, granulles 1–2 mm; Critéria Ind. e Com. de Produtos Medicinais e Odontológicos Ltda., São Carlos, Brazil) or the control test with Bio-Oss® (Bio-Oss® Large, granulles 1–2 mm; Geistlich Pharma AG, Wolhusen, Switzerland) was determined randomly, using a toss of coin, as previously decribed [6]. This study was approved by the Institutional Review Board. All patients were given written information about the study, and their consent was registered in their charts. The primary outcome of this study is histomorphomety evaluation and radiographic analysis. Secondary outcomes are additional outcomes from clinical implant survival.

### Patients selection

Sample calculation was based on the following two questions: How much of new bone formation could interfere with the clinical outcome? What is the amount of remaining particles of graft material that can interfere with the clinical outcome? There are no precise answers in the literature on this topic; however, it is believed that a small amount of new bone (less than 15%) and a large amount of remaining particles (above 40%) may interfere with the osseointegration process. The medium values for variables in the literature range from 12 to 69% and 14 to 60%. Thus, from the data available for Bio-Oss in the literature, the minimum number of 10 subjects generates power test of 0.80, as most of the studies in the literature with this methodology (split-mouth). Thus, we chose to start our study with 15 individuals. Volunteers with age between 39–70 years old were selected among those patients that were referred for implant dentistry program at Division of Oral and Maxillofacial Surgery of Piracicaba Dental School, State University of Campinas, Brazil, from October 2012 to December 2014. Inclusion criteria included good general and physical health, good oral health, nonsmoker, no active periodontitis, residual alveolar bone height ≤ 4 mm, and need for two-stage sinus augmentation. Patients with compromised general health (ASA III or IV-American Society of Anaesthesiology [11], drug abuse, maxillary sinus pathology (chronic sinusitis) and preexisting sinusal procedures/disease were excluded. After clinical and radiographic evaluation, through orthopantomography and CBCT, fifteen patients were recruited and provide informed consent.

### Surgical procedure for sinus lift

**Fig. 1:**
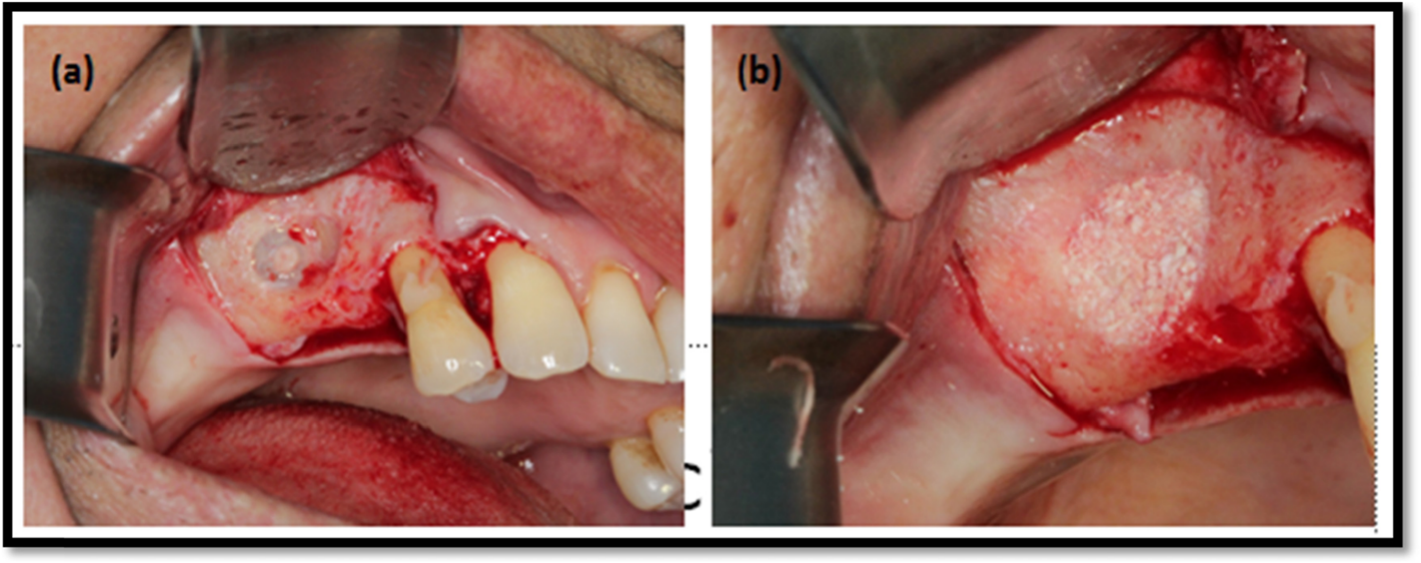
Midcrestal and vertical buccal incisions were made along the residual alveolar bone. A diamond bur is used to create a window and placed deproteinized bovine bone (control or test) into the maxillary sinus

A two-stage approach was applied. In the first stage, the maxillary sinus floor was augmented bilaterally, carried out according to Tatum [12]. Surgery (Fig. 1a) was performed by the same surgeon (DRG) simultaneously on both sides under local anesthesia (lidocaine 2% + epinephrine 1:100.000; DFL Ind. e Com. SA, Rio de Janeiro, Brazil) and antibiotic prophylaxis (amoxicillin 1 g, 1 h before surgery, and 500 mg every 8 h for 7 days). The patients used a mouth rinse (chlorhexidine 0.12%) 60 s immediately prior to surgery and every 12 h during 7 days.

Full-thickness flaps were elevated, followed by the use of a round diamond bur under irrigation with sterile saline to create an bone window. Schneiderian membrane was carefully elevated and the bone window was pushed inside the cavity. Sufficient material was placed to fill the cavity was achieved (Fig. 1b), the same amount of material in placed in both maxillary sinuses. The space created between maxillary alveolar process and the new sinus floor was carefully packed with bone graft particles. Resorbable collagen membrane (GenDerm, Baumer SA, Bauru, SP, Brazil) was used when perforation of Schneiderian membrane occurred. Sutures (Ethicon, São Paulo, Brazil) were removed after 7 days. Patients were followed up at 7, 15, 30, and 90 days postoperative. Then the implants were installed after 6 months following sinus floor augmentation.

### Radiographic analysis

**Fig. 2:**
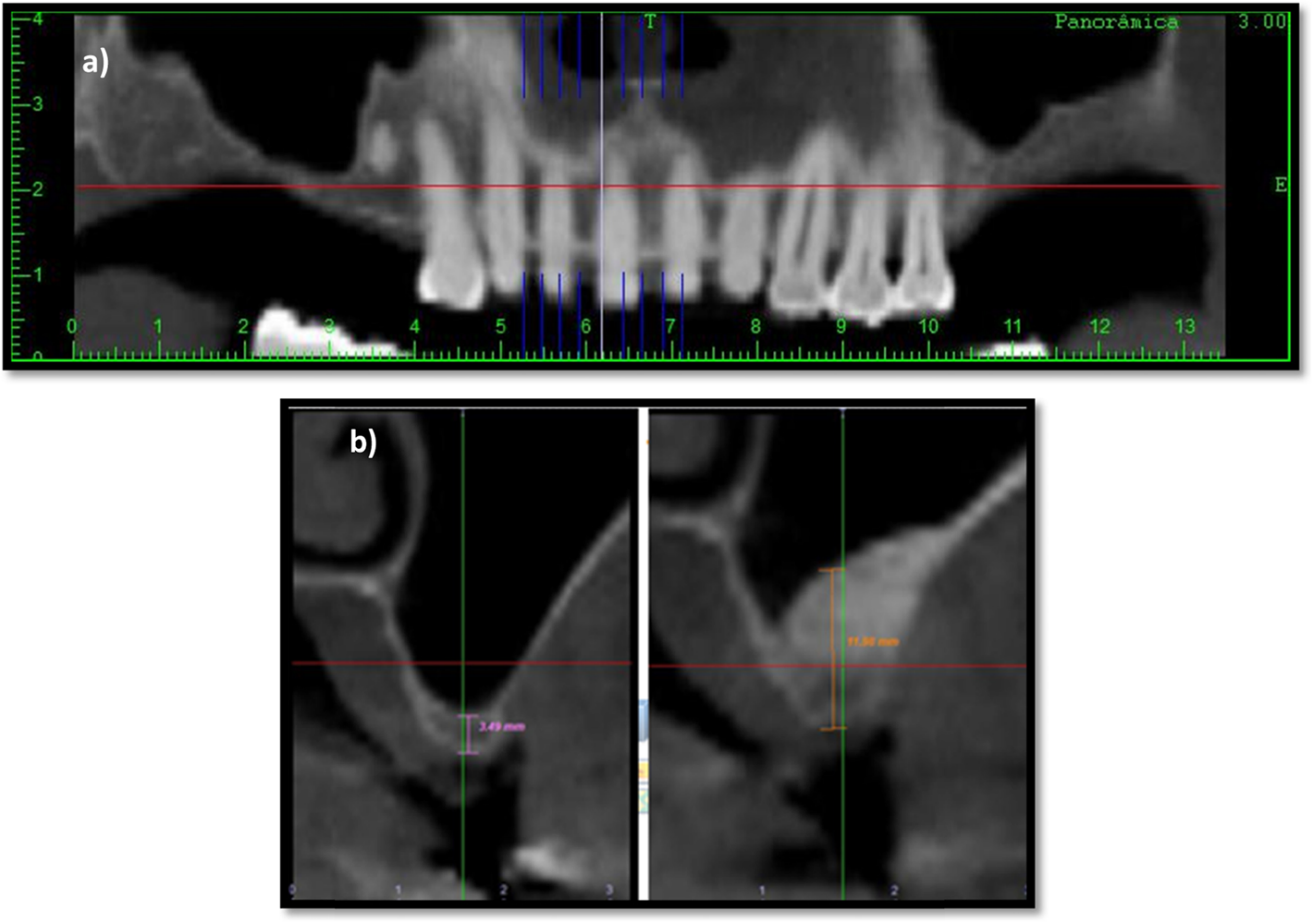
The preoperative (**a**) and postoperative (**b**) images were used to evaluate the measurements of bone ridge height using a software tool of DentalSlice®

CBCT was used to evaluate the sinus health, morphology, and residual alveolar bone height. For all patients, radiographic assessments were recorded preoperatively (Fig. 2a) and at 6 months after SL by the same examiner. The CBCT analysis was performed using a software program (DentalSlice, Bioparts, Brasília, DF, Brazil) at the point of greatest resorption of the bone tissue added to the mesial/distal adjacent slices of the CBCT and averaging between them (Fig. 2b). Six months after SL, the point of greatest augmentation of bone tissue was recorded added to the mesial/distal adjacent slices of the CBCT and averaging between them.

### Dental implant surgery and biopsy retrieval

**Fig. 3:**
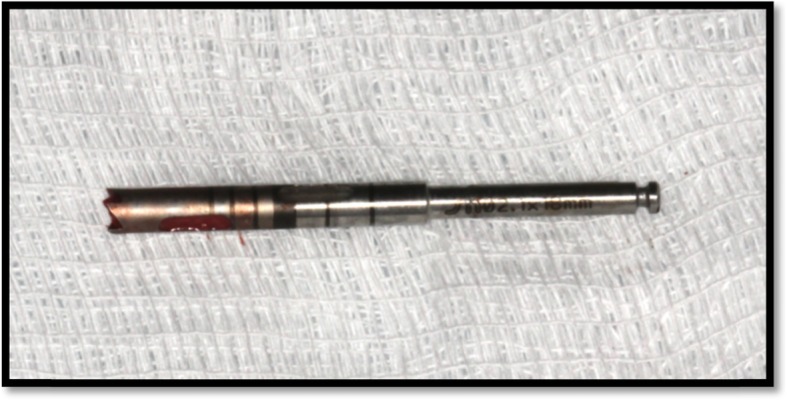
Bone taken with a trephine burr for biopsy

**Fig. 4:**
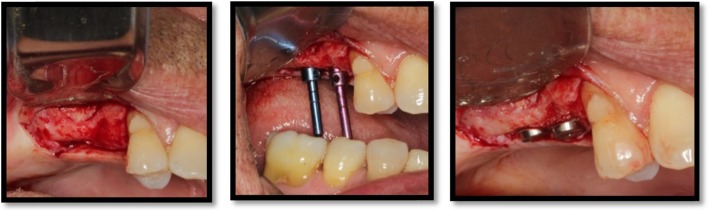
Implants placed in the grafted sinuses after 6-month healing

Six months after SL, biopsy specimens were obtained under local anesthesia (lidocaine 2% + epinephrine 1:100.000) and a full-thickness flap using a 2-mm internal diameter trephine drill (2.0 × 18 mm/external diameter of 2.8 mm; Sistema de Implantes Nacional, São Paulo, Brazil) under sterile saline irrigation. A perforation of the Schneiderian membrane was accepted, similar to other study [13]. Bone biopsies were made in the position by trephine bur in the coronal-apical direction by crestal approach, approximately 7 mm depth, as used in other study [14]. One lateral (horizontal) biopsy was taken from each augmented sinus (Fig. 3). The biopsies were used for bone histology and histomorphometric analysis. Implants were installed (Fig. 4) into the trephined holes and were left an average of 6 months prior to definitive prosthetics and loading.

### Histologic preparation and histomorphometric analysis

The bone specimens were left inside the trephine in order to preserve the bone structure for histological examination. An apicoronal orientation was maintained so that the bone analyzed was more in the apical area. The trephine handle was used as a reference for apicoronal orientation, and the orientation was marked on each histological section.

The bone biopsies were immediately fixed in 10% formalin (pH 7), followed by dehydration using an ascending series of alcohols (70%, 80%, 96%, and 100%). Each sample remained 48 h at each concentration. After this process, the samples were embedded in resin (LR White resin, London Resin Company, London, UK) and kept under stirring for 60 min. Subsequently, the specimens were stored and maintained for at least 12 h at a temperature of 4 °C. After this period, materials were kept in a vacuum for 1 h, agitated for equal time, and again stored in a refrigerator for 24 h. This routine was repeated for 15 days, changing the resin every 48 h. On the 15th day, the parts were identified and brought to the oven at 60° to induce resin polymerization. The specimens were bisected longitudinally using a saw-precision cutting band 0.1 mm/D64 (EXAKT system, Norderstedt, Germany) and after, sanded and polished on sandpaper and polishing cloths of different granulation starting at 320, followed by 800, 2500, and 4000 (Hermes Abrasives Ltd, Virginia beach, VA, USA). From each trephine, two pieces (slices) with about 70 μm, and the best one was chosen for analysis and subject to staining using Stevenel’s blue and Alizarin red method [15].

**Fig. 5:**
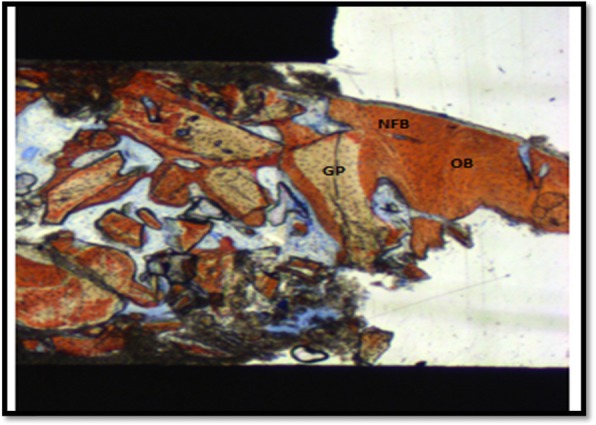
Image representing the histological findings observed in ROI in the control sites. ROI represents the area from the junction of the native bone (i.e., the former floor of the sinus cavity) and ends until the frame delimited into the sample. The “old bone” (OB) was evidenced as a lamellar bone area, while the new formed bone area (NFB) was characterized as a parallel-fibered bone with areas of interlaced fibers. The residual graft particles (GP) were present in close contact to the new formed bone, evidencing a good osseointegration rate

**Fig. 6:**
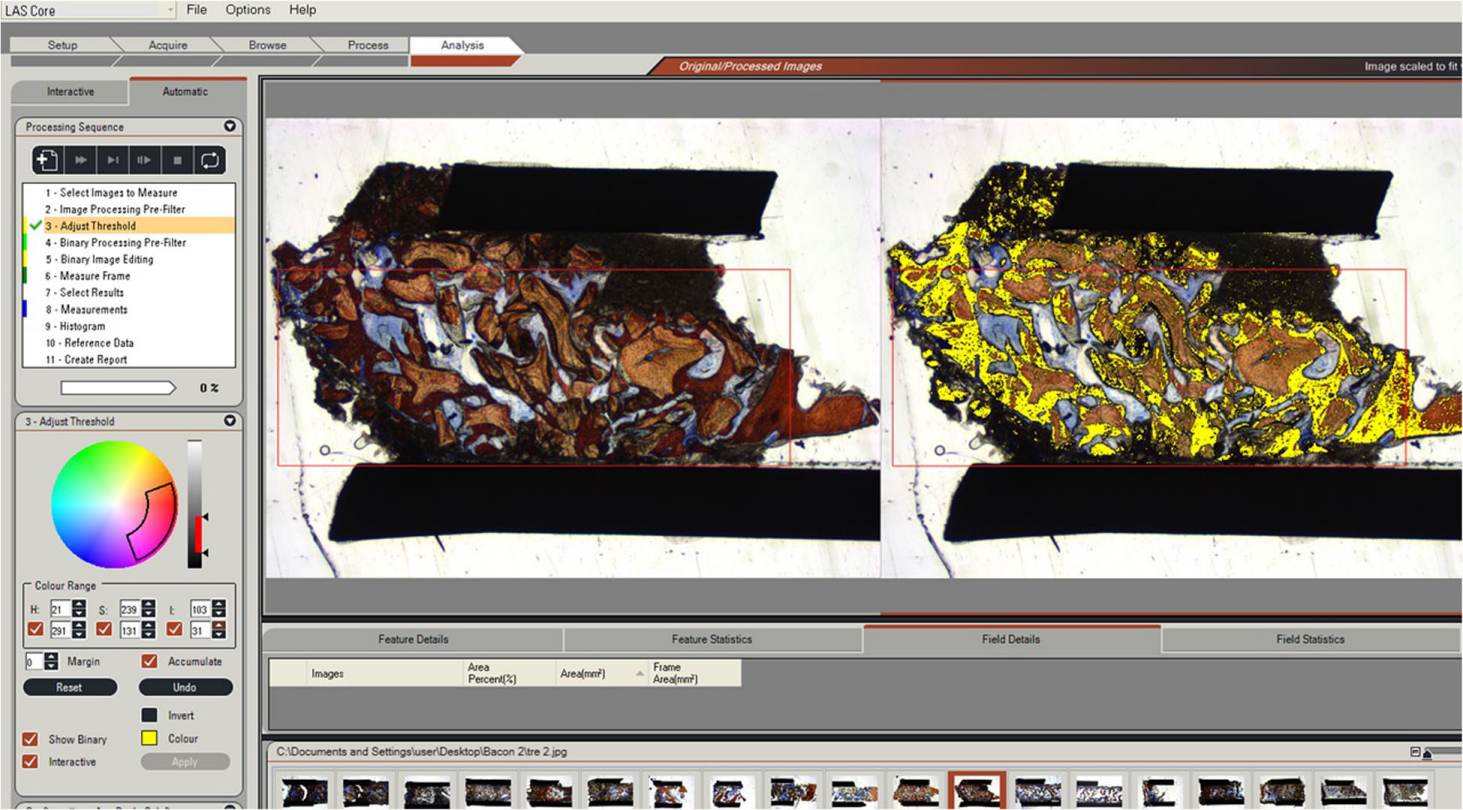
A part histological image highlighting the area of new bone (%NB—yellow color), residual graft particles (%RGP—orange color), and connective tissue (blue color) within a region of interest allowing then the calculation of the relative fraction (%) of each parameter

**Fig. 7:**
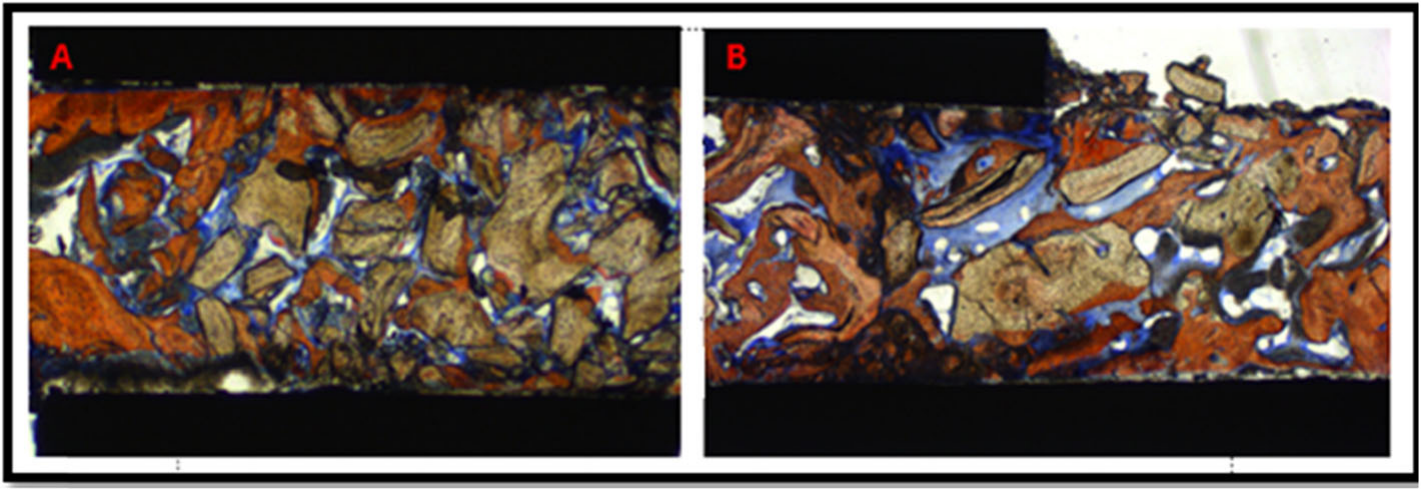
Schematic image illustrating the histomorphometric linear measurements in the control (**a**) and test (**b**) groups

Histomorphometric measurements were performed by experts following the routine technique. The measurements were carried out at ×100 magnification. The examiner was blinded and carried out using a specialized histomorphometric analysis software program—LEICA DMLB Microsystems microscope (Leica Microsystem, Wetzlar, Germany) outfitted with a LEICA DC300F digital camera (Leica Microsystems). Sections stained with Stevenel’s blue and Alizarin red were used for the analysis. The region of interest (ROI) was the tissue formed above native bone; it was delineated after carefully studying to observe the margin between the residual alveolar bone and the augmented bone of the whole biopsy. This ROI (Fig. 5) represented the area from the junction of the native bone to the apical limit of the biopsy (Fig. 6). The digitalized images (control and test) were analyzed through the LAS v. 4.1 computer software (Leica Microsystem Image Solutions, Wetzlar, Germany) by the same examiner (HFS) with knowledge of the experimental groups (Fig. 7). The bone area measurements evaluated the percentages of mineralized bone and residual graft particles in relation to the percentages of connective tissue [16]. The definitions of each parameter are as follows:

- %NB: area of newly formed bone/area
- %GP: area residual graft particles/area
- %CT: area of the connective tissue
- %TB: area of total bone

### Blinding and statistical analysis

The researchers who performed the statistical analysis, radiographic, and histomorphometric evaluation were blinded to treatments. Radiographic parameters (vertical bone height) were measured at baseline (preoperative) and 6 months after surgery procedure of SL (postoperative). Quantitative data was recorded as the mean value ± SD. The conformity of the parameters to the normal distribution was assesses by the Shapiro-Wilk test. Paired sample *t* test was used for the intragroup comparisons of the parameters with normal distribution. ANOVA two-way test was used for the intergroup comparisons of parameters with normal distribution. A *p* ≤ 0.05 was considered to represent statistically significant differences between control and test groups.

## Results

### Clinical follow-up and radiographic findings

Fifteen patients were included in this research; one patient was lost of the study during the implant insertion period and a second patient was excluded from data analysis because the bone biopsy resulted non-feasible for histomorphometric analysis due to disruption of the material during the preparation of the specimens.

**Fig. 8:**
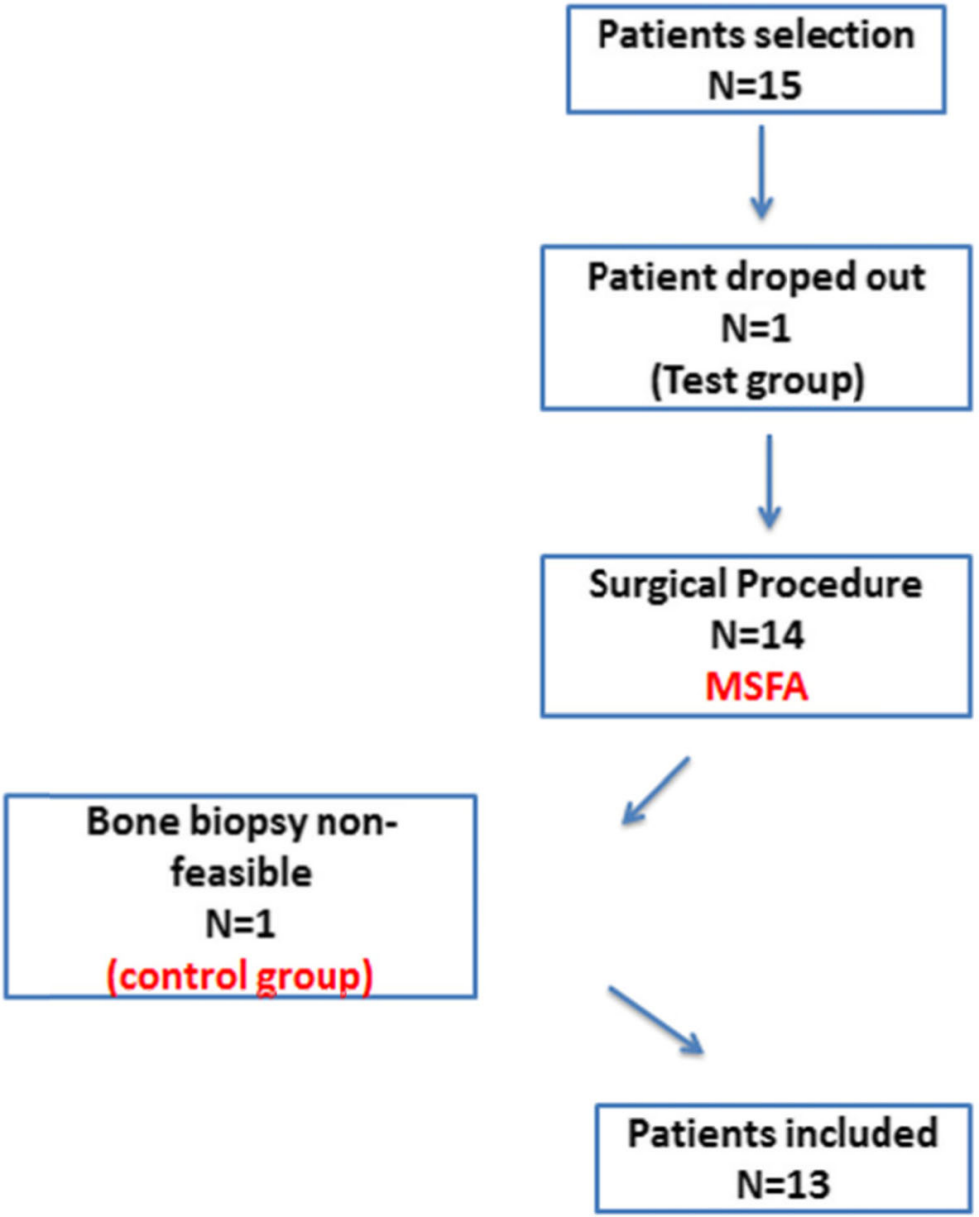
Schematic image illustrating the evaluation about the number of patients

Thirteen volunteers were included in this study, 7 women and 6 men, with age 55.0 ± 8.13 years old. Three patients presented hypertension, three hypothyroidism, and two depression. All of them received medical treatment and follow-up. The Fig. 8 shows the evolution of patients.

**Fig. 9:**
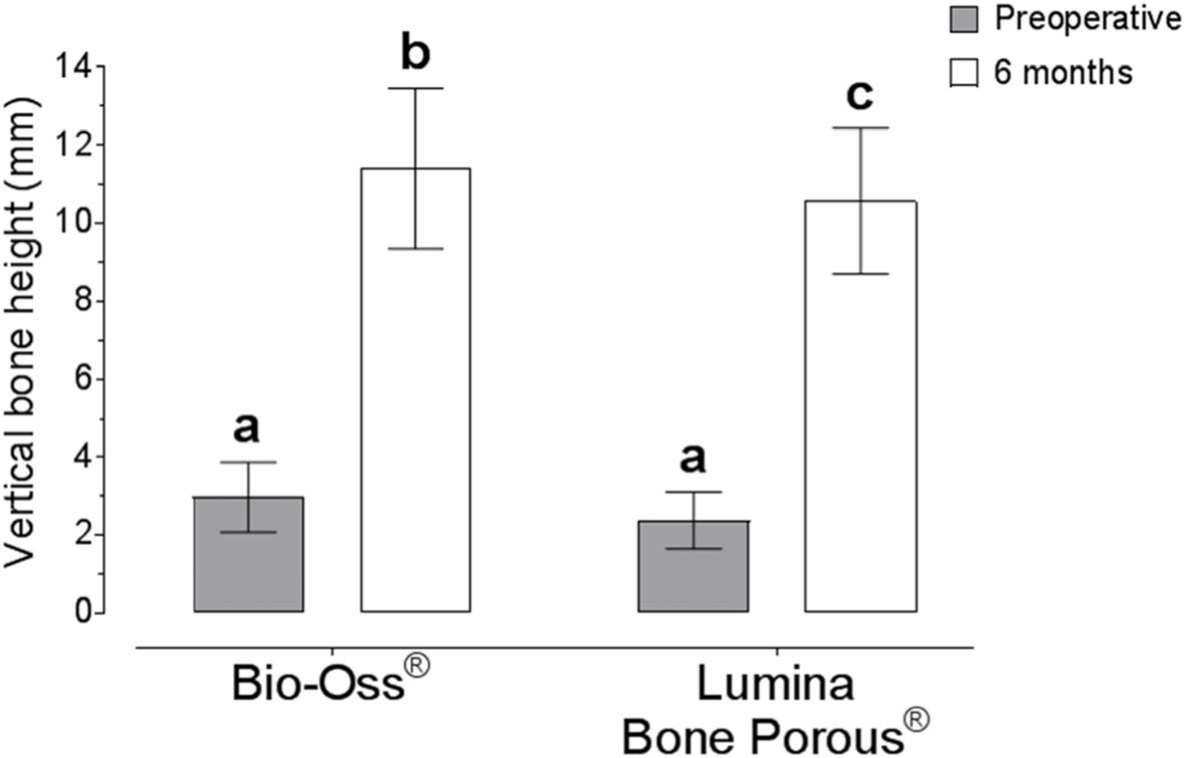
Measurements demonstrate the radiographic findings (vertical bone height) preoperative and postoperative after MSFA. ANOVA two-way was used to compare intergroup

The radiological results demonstrated that, prior to sinus grafting, the mean resident bone ridge height in the deepest portion of maxillary sinuses floor was 3.11 ± 0.83 mm in the Bio-Oss® and 2.38 ± 0.75 mm in the Lumina-Bone Porous® group. Six months after surgery, alveolar ridge height were 11.56 ± 2.03 mm in the Bio-Oss® and 10.62 ± 1.93 mm in the Lumina-Bone Porous®. These results showed normal distribution, both Lumina-Bone Porous® and Bio-Oss group when the Shapiro-Wilk test was used. The tomographic findings demonstrated that both ABB resulted in bone height at 6 months favorable the implants placement. There were no difference between the preoperative period for the groups (*p* = 0.112). The increase in alveolar bone height scores was significant between pre-augmentation and 6 months after SL in both groups (*p* < 0.0001). Intergroup differences were found after SL to be statistically significant for the groups (ANOVA two-way, Tuckey test *p* = 0.020) (Fig. 9).

In four subjects were observed an intraoperative perforation of Schneiderian membrane. These perforations were covered with and absorbable collagen membrane. None of these patients experienced postoperative complications. Furthermore, two patients showed postoperative sinusitis in left maxillary sinus (1 control and 1 test group); these were successful treated with oral antibiotics (amoxicillin 875 mg and clavulanate potassium 125 mg) c/12 h for 7 days and saline nasal irrigation five times a day for 7 days, without other complications.

Thirty-three implants were inserted in augmented areas (External-Hex implants 4.0 × 9,0 mm, Neodent-Jjgc Industria e Comercio de Materiais Dentarios S.A, Curitiba, PR, Brazil), and 56 in other sites (Table 1) and there were no statistically significant differences (Fisher, *p* = 0.495). In one patient was not possible to insert an implant in the second stage due to the lack of primary stability (Bio-Oss® group). Two patients experienced late implant loss in the augmented area (Lumina-Bone Porous®). The survival rate for augmented area was 93.9% and the patients were under revision at 3-year follow-up after final prosthetic restoration.

**Table 1:** Information data of implants survival about Bio-Oss® and Lumina-Bone Porous® (*n*)

	Bio-Oss® (*n*)	Lumina-Bone Porous® (*n*)	Total
Implant installed	**15**	**18**	**33**
Implant loss	**0**	**2**	**2**
Survival rate (%)	**100**	**88.88**	**93.93**

### Histologic and histomorphometric findings

**Fig. 10:**
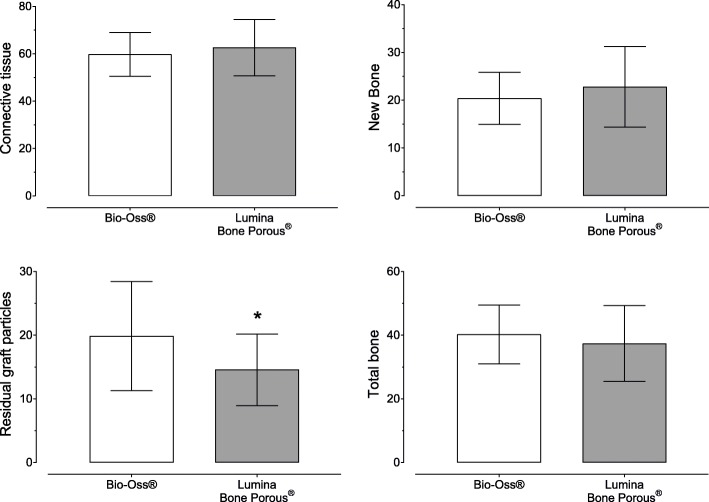
Histomorphometrical analysis results. Difference between Bio-Oss and Lumina-Bone Porous about newly formed bone, residual graft particle, connective tissue, and total bone. The symbol above the result demonstrates statistical significance

The bone specimens of each volunteer were kept inside the trephines during histological processing. A total of 26 samples and 52 slices were obtained in our research regarding to 13 patients who conclude to all inclusion criteria. In summary, trabecular bone with woven and lamellar architecture was observed bridging the graft particles in all samples and no inflamatory reactions could be observed. The Shapiro-Wilk test showed normal distributions in the two groups; a paired *t* test was used (Fig. 10) with no differences (*p* = 0.40) between the Bio-Oss® (20.4 ± 5.4%) and Lumina-Bone Porus® (22.8 ± 8.5%) groups in accordance to the amount of newly formed bone. The amount of non-resorbed material is showed and it was observed that the Bio-Oss ® group (19.9 ± 8.6%) had a higher amount of residual graft particles than Lumina-Bone Porous® group (14.6 ± 5.6%), showing differences (*p* = 0.015). The connective tissue observed in the Bio-Oss® (59.74 ± 9.2) and for Lumina-Bone Porus® group (62.61 ± 11.8) shows no differences between them (*p* = 0.174) and the total bone showed in the Bio-Oss® (40.25 ± 9.2) and in the Lumina-Bone Porus® group(37.38 ± 11.8) no differences (*p* = 0.174).

## Discussion

In recent years, several studies have been performed to get the best technique searching regulatory mechanisms used in implant dentistry [17, 18] and reconstructive oral surgery [19, 20]. Implant placement in the edentulous posterior maxilla is often limited due to bone loss and increasing pneumatization of the maxillary sinus [1, 2, 21]. Augmentation procedures and materials used for the posterior maxillary reconstruction have been described and established since 1980 [12]. In our study, we aimed to compare clinical, radiographic, and histomorphometric features of two different composites used in SL. In this case, our method was used Bio-Oss® or Lumina-Bone Porous® isolated, without mixture of graft material with autologous bone (AB), in a randomized perspective, using a regular method [9, 13]. Some researchers have been used the mixture of graft material with AB [20, 22], because autogenous bone as the sole material for sinus augmentation provides the unique benefits of osteoinductivity, osteoconductive, and osteogenic potencial [19]; however, extensive remodeling may occur [23] added the disadvantages of donor site and possibility of surgical complications [24].

Considering a low number of implants (33 implants), there were comparable survival rates (total augmented area was 93.9%) with the international review [21, 24]. Xavier et al. [4] in the a split-mouth randomized study including 15 patients, using autogenous or fresh frozen bone for SL, showed good regenerative capacity in their clinical findings (eighty implants were inserted, two implant failures). Schmitt et al. [19], after a mean time in function of 5 years, founded the implant survival was 93.75% in the ABB and 92.86% in the ABB + AB (ratio of 1/1) group. On the other hand, Sverzut et al. [25] selected 10 adult patients and analyzed the reconstructive capacity of calcium phosphate cements in SL and their results showed no evidence of resorption/substitution of biomaterial. However, during instrumentation and installation of dental implants, it was observed that the abundant presence of material, which was friable, aborting the implant placement in some cases. It was concluded despite the osteoconductive capacity, this material does not support sufficient amount of new bone formation in SL and dental implant placement.

CBCT analysis is performed to assess quantity and quality of bone formation; the mean resident bone ridge height in the deepest portion of maxillary sinuses floor was 3.11 ± 0.83 mm in the control group and 2.38 ± 0.75 mm in the test group, close to other research [26]. In our study, after SL the Bio-Oss® group shows bone ridge height of 11.56 ± 2.03 mm and Lumina-Bone Porous® of 10.62 ± 1.93 mm. Of course, after SL surgery, there were differences between the initial and amount of bone height gained (*p* < 0.50), in accordance with other studies [7, 19, 20, 22], supporting that both materials can be successfully used for reconstructive procedures in SL.

In terms of histological and histomorphometrical outcome, it is possible to observe that Lumina-Bone Porous® material exhibits similar Bio-Oss® performance in NB (22.8 ± 8.5% vs 20.4 ± 5.4% respectively, *p* = 0.40) and substitution was more rapidly than Bio-Oss® (14.6 ± 5.6% vs 19.9 ± 8.6% respectively, *p* = 0.015). It is possible to speculate about the particle size as influence the condition of biomaterial substitution and the relation with the environment related to bone metabolism, as proved by Kluppel et al. [27], even when both materials have the same particle size, compaction, and proportion in different areas of the graft could get different ratios in particle maintained in the short and long time. However, considering the same structural conditions and bone volume obtained in the both groups under SL with AAB, we can expect the favorable evolution in the long time because the implant load could permit a favorable response and stimulation on the bone in contact. Biopsies obtained in other research after SL with Bio-Oss® revealed NB of 25% after 3 to 5 months [28] and 35% after 6 to 8 months [28, 29], close to this results.

Stefano et al*.* [30] conducted an histomorphometric study comparing ABB and enzyme-deantigenic equine bone (EDEB). It was found a greater percentage of NB in EDEB group (46.86% versus 25.12%). However, in the clinical point of view was not found a difference between the groups during a follow-up of 3 years. Calasans-Maia et al*.* [9] performed a clinical study with 20 volunteers to compare two ABB (Bio-Oss® and Osseous®). Both materials were biocompatible and promote osteoconduction. The use of these materials led to successful dental implants after 6 months of successful grafting without the need for AB. The efficacy of xenografts as a sinus bone replacement graft may be due to a combination of factors related to biomaterial quality and related to local conditions as showed by Parra et al*.* [31, 32], who incorporate variables as sinus membrane, bone quality, and presence of teeth in the anterior or posterior position as important for new bone formation.

Bio-Oss® has been shown to be a biologically inert osteoconductive composite and the most evaluated for SL [9, 26], considering the use to have equal results when compared to autogenous graft [19, 22] in some techniques; Bio-Oss® has been considered as the gold standard of the xenografts [33]. On the other hand, Lumina-Bone Porous® is a new composite from the same origin that provides further correlations. Both xenograft materials used for SL show no differences in term of NB (*p* > 0.05), show clear differences between the initial stage and 6-month follow-up high bone level (*p* < 0.001), and no inflamatory reactions could be observed. The neoformation of mineralized bone occurred predominately around and adjacent to the bovine bone substitute particles and adjacent to the residual ridge maxillary bone, separated by a resorption-apposition line. It is important to note that despite the higher percentage result of the test group in relation to the NB, the TB volume was higher in the control group (40.25 ± 9.2 vs 37.38 ± 11.8). Residual graft particles were partially covered by connective tissue and some were close to newly formed bone, founded statistically significance measurements (*p* < 0.05). These histologic features were observed in both groups of this study, showing good predictability, and offering stability for the dental implants surgery, despite physical and chemical differences in the preparation of the composite.

This study presents some limitation related to split-mouth design, even though individual variables are minimized, the right and left maxillary sinuses present anatomical differences. In addition, this study had a limited sample and a short follow-up time after prosthetic rehabilitation. Regarding histological analysis, despite the biopsy technique was performed in order to keep the bone inside the trephine, some material could be lost.

## Conclusion

We conclude that the null hypothesis is probably correct, and it should be accepted in favor of the alternative hypothesis. Both materials Bio-Oss® and Lumina-Bone Porous® can be used in the maxillary sinus floor augmentation with good predictability in clinical, radiographical, and histological point of view. Future studies with more patients correlating different properties in maxillary sinus floor augmentation procedure like long-term effects on sinus bone grafts and dental implants are needed.

## Abbreviations

AB: Autologous bone
ABB: Anorganic bovine bone
CBCT: Cone beam computed tomography
GP: Residual graft particles
NB: Newly formed bone
SL: Sinus lift
